# Functional iron deficiency in patients on hemodialysis: prevalence, nutritional assessment, and biomarkers of oxidative stress and inflammation

**DOI:** 10.1590/2175-8239-JBN-2018-0092

**Published:** 2019-08-22

**Authors:** Juliana Carvalho Romagnolli Plastina, Vitor Y. Obara, Décio Sabbatini Barbosa, Helena Kaminami Morimoto, Edna Maria Vissoci Reiche, Andrea Graciano, Vinicius Daher Alvares Delfino

**Affiliations:** 1 Universidade Estadual de Londrina, Centro de Ciências da Saúde, Londrina, PR, Brasil.; 2 Universidade Estadual de Londrina, Departamento de Patologia, Análises Clínicas e Toxicológicas, Londrina, PR, Brasil.; 3 Hospital Evangélico de Londrina, Londrina, PR, Brasil.; 4 Universidade Estadual de Londrina, Departamento de Clínica Médica, Londrina, PR, Brasil.

**Keywords:** Renal Insufficiency, Chronic, Anemia, Iron-Deficiency, Oxidative stress, Inflammation, Nutrition Assessment, Insuficiência Renal Crônica, Anemia Ferropriva, Estresse oxidativo, Inflamação, Avaliação Nutricional

## Abstract

**Introduction:**

Anemic patients with chronic kidney disease (CKD) can be divided into anemic patients without or with functional iron deficiency (FID). The increase in the number of cases of hemosiderosis in patients on hemodialysis (HD) attributed to excessive intravenous iron replacement has called for the investigation of the factors involved in the genesis of FID.

**Objectives:**

This study aimed to describe the prevalence of FID in patients with CKD on HD, characterize the included individuals in terms of clinical and workup parameters, and assess their nutritional, oxidative stress, and inflammation statuses. This cross-sectional study assembled a convenience sample of 183 patients with CKD on HD treated in Southern Brazil. Patients meeting the inclusion and exclusion criteria were divided into two groups, one with anemic subjects with FID and one with anemic patients without FID. Participants answered a questionnaire probing into socio-epidemiological factors, underwent anthropometric measurements, and were tested for markers of anemia, oxidative stress, inflammation, and nutrition.

**Statistical analysis:**

The date sets were treated on software package GraphPad InStat version 3.1. Variables were tested with the Kolmogorov-Smirnov, chi-square, Student’s t, and Mann-Whitney tests. Statistical significance was attributed to differences with a *p* < 0.05.

**Results:**

Markers of inflammation were not statistically different between the two groups. Markers of anemia and nutrition were significantly lower in patients with FID. Patients with FID were prescribed higher doses of parenteral iron (*p* < 0,05).

**Discussion:**

FID was associated with lower nutritional marker levels, but not to increased levels of markers of inflammation or oxidative stress, as reported in the literature. Additional studies on the subject are needed.

## Resumo

**Introdução:**

A anemia na DRC pode ser dividida em anemia sem deficiência funcional de ferro e com deficiência funcional de ferro (ADFF). Diante do aumento dos casos de hemossiderose em pacientes em hemodiálise, atribuídos à reposição excessiva de ferro endovenoso, maiores conhecimentos sobre os fatores envolvidos na gênese da ADFF são importantes.

**Objetivos:**

documentar a prevalência de ADFF em renais crônicos em hemodiálise. Caracterizar clínica e laboratorialmente os portadores de ADFF em HD e avaliar o estado nutricional, estresse oxidativo e inflamatório. Estudo transversal, amostra de conveniência, envolvendo 183 renais crônicos em hemodiálise no sul do Brasil. Após aplicação dos critérios de exclusão, os pacientes foram separados em dois grupos: portadores de anemia com e sem deficiência funcional de ferro. Foram submetidos a questionário socioepidemiológico, à análise antropométrica e análise laboratorial dos marcadores de anemia, estresse oxidativo, inflamatórios e nutricionais.

**Análise estatística:**

programa GraphPad InStat versão 3.1. Foram aplicados os testes: Kolmogorov-Smirnov, qui-quadrado, t de Student e Mann-Whitney. Nível de significância adotado de 5%.

**Resultados:**

não houve diferença significativa nos marcadores inflamatórios entre os dois grupos. Houve diferença significativa nos marcadores de anemia e nutrição, significativamente menores nos pacientes com ADFF. Pacientes com ADFF receberam doses mais elevadas de ferro parenteral (*p* < 0,05).

**Discussão:**

ADFF esteve associada a menores valores de marcadores nutricionais, mas não esteve associada a marcadores inflamatórios ou de estresse oxidativo aumentados, como relatado na literatura. Estudos adicionais sobre o tema são necessários.

## Introduction

Anemia appears in the early stages of chronic kidney disease (CKD) and prevalence increases as renal function deteriorates. Oral or parenteral iron therapy and erythropoiesis-stimulating agents (ESA) are relevant elements in the care provided to patients with CKD, since anemia is one of the primary factors in the etiology of cardiovascular death in this group of patients.^1^

The etiology of anemia in patients with CKD is multifactorial. Primary contributing factors include absolute or functional iron deficiency; relative erythropoietin deficiency; deficiency of micronutrients such as folic acid and complex B vitamins; chronic inflammation; infection; blood loss after extracorporeal circulation; blood collected for workup purposes; and hemolysis.^2^ Chronic inflammation has been associated with decreased survival of patients with advanced-stage disease. Serum C-reactive protein (CRP) and proinflammatory cytokine levels - including tumor necrosis factor alpha and interleukins 1 and 6 - are increased in 30-50% of the patients with CKD.^1^^,^ 2 ^,^ 3 IL-6 increases serum levels of hepcidin, a liver-derived peptide hormone that inhibits the duodenal absorption of iron and the mobilization of iron in the reticuloendothelial system.^2^ High hepcidin levels lead to iron sequestration and hypoferremia. Inflammation has also been implicated in decreased iron bioavailability for erythropoiesis and low albumin levels, a sensitive marker of malnutrition.^3^^,^ 4

Although the guidelines for the treatment of anemia in patients with CKD favor a relatively liberal use of intravenous iron with the purpose of strengthening the action of erythropoietin (EPO), they fail to consider that high ferritin levels may induce hemosiderosis. Intravenous iron is prescribed to patients with ferritin levels ranging between 500 ng/dL and 1200 ng/dL. In healthy individuals, these levels might be indicative of hemosiderosis.^5^

This study aimed to describe the prevalence of FID in patients with CKD on HD, characterize the included individuals in terms of clinical and workup parameters, and assess their nutritional, oxidative stress, and inflammation statuses.

## Materials and methods

### Design and population

**Figure 1:**
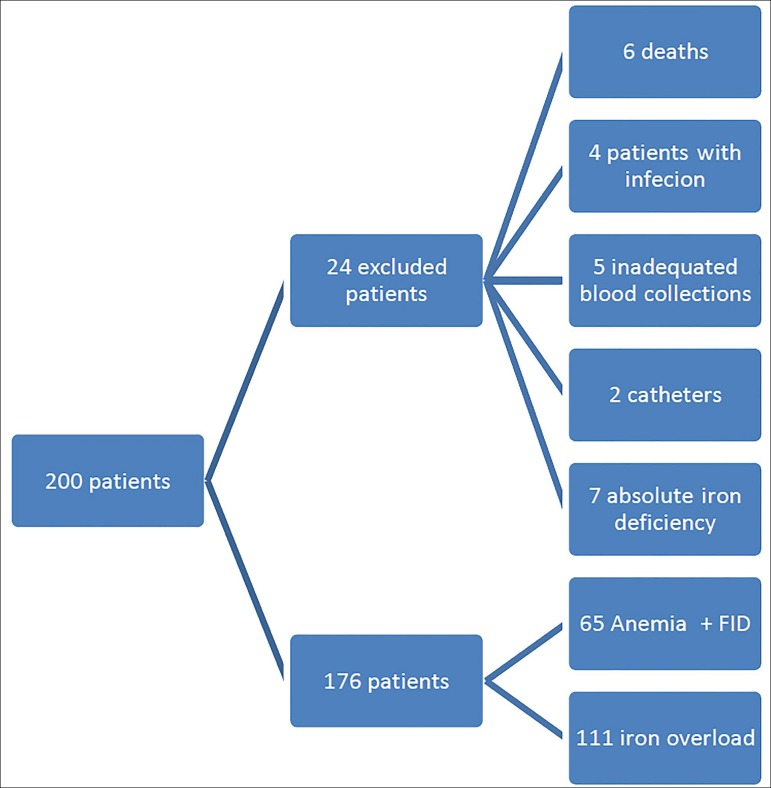
Horizontal Flowchart.

This cross-sectional study was carried out in two hemodialysis units in Southern Brazil. Patients aged 18 years or older on hemodialysis with native arteriovenous fistulae or grafts for at least three months were included in the study in June 2014. Individuals with temporary hemodialysis catheters, cancer, active infection or infection that caused hospitalization within 15 days of blood collection, hepatitis B or C, or infection by the human immunodeficiency virus were excluded. Two hundred patients met the inclusion criteria of the study. Twenty-four were excluded, four for having active infection, five due to inadequate blood collection, two for requiring hemodialysis catheters, seven for having anemia with absolute iron deficiency, and six for having died (Figure 1).

The remaining patients were divided into two groups, one with 65 patients with anemia and FID characterized by ferritin levels > 200 ng/dL and percent transferrin saturation < 20%, and another with 111 patients without FID and with iron overload and ferritin levels > 500ng/dL as per the guidelines of the KDOQI.

The patients underwent dialysis in sessions lasting for 210-240 minutes three times a week, with blood flow rates of 300-450 mL/min and dialysate flow rate set at 500 mL/min. Hemodialysis was performed with medium flow polysulfone membrane filters, with a surface area matched to the body surface area of each patient. The water used in the hemodialysis sessions met the national criteria for water quality. The non-equilibrated target Kt/V was set at 1.2. Anemia was managed in accordance with the KDOQI guidelines. Patients were prescribed iron therapy with ferric hydroxide 100 mg/ampoule. Participants were provided ample information on the study and gave written consent before joining the study. The Research Ethics Committee of the Londrina State University approved the study.

### Data collection

Patient charts and interviews were used as sources of demographic and anthropometric data, in addition to information on time on dialysis, comorbidities, and prescribed medication. Mean doses of erythropoietin and iron were calculated based on the prescriptions issued within 30 days of blood collection, with values described as U/kg/month and mg, respectively. Blood was collected before patient heparinization. Patients were asked about whether they smoked or drank alcohol. Race/ethnicity was self-reported.

### Anthropometric measurements

The patients were asked to wear light clothing to have their dry weight (weight patients had at the end of hemodialysis sessions while feeling well and without edema) measured on a Filizzola scale with a readability of 0.1 kg. Patients were asked to stand against a wall with feet together flat on the ground and had their height measured with a measure tape. Height was reported based on the nearest centimeter. The height of patients unable to undergo the measuring process was self-reported or reported by a relative. The body mass index (BMI) was calculated as weight divided by height in meters squared.

Waist circumference was measured to the nearest centimeter with a flexible plastic tape measure placed in the midpoint between the lowest rib and the anterior-superior portion of the iliac crest with the patient standing. The same plastic tape measure was used to measure the mid-upper circumference of the dominant arm (patients with an arteriovenous fistula in this area of the arm had the circumference of the non-dominant arm measured).

### Biochemical analysis

Biochemical analyses were performed at the laboratory of the Londrina University Hospital, a member of quality control programs PELM and PNCQ. The patients were advised to fast for eight hours prior to blood collection. Patient blood samples of approximately 20 mL were collected in anticoagulant-free vacuum tubes (Vacutainer^®^, Franklin Lakes, NJ, USA) during venipuncture at the start of the first hemodialysis session of the week. Intravenous iron was discontinued seven days before the collection of blood samples. The samples were centrifuged at 3000 rpm for 15 minutes. Blood serum was stored at -70ºC until the tests for biomarkers of inflammation and oxidative stress were carried out. Serum creatinine, urea before and after hemodialysis (to calculate dialysis adequacy - Kt/v), serum iron, ferritin, transferrin saturation, hematocrit, hemoglobin, parathyroid hormone (PTH), calcium, phosphorus, alkaline phosphatase, and albumin levels were measured following standard laboratory practices. The intra and inter assay coefficients of variation were less than 5% for all analytes.

### Inflammation parameter measurement

Inflammation parameters were measured based on interleukin-6 (IL-6) and CRP levels.

Serum high-sensitivity CRP (hs-CRP) levels were determined by turbidimetric assay (ARCHITECT c8000, Architect, Abbott Laboratory, Abbott Park, IL, USA). The intra and inter assay coefficients of variation were less than 5%.

IL-6 levels were quantified with the aid of a commercially available kit from eBioscience. Results were expressed in pg/mL of serum. The intra and inter assay coefficients of variation were less than 5%.

### Oxidative stress measurement

The following panel was used to measure oxidative stress: levels of nitric oxide metabolites - NOx; quantification of advanced oxidation protein products - AOPP; paraoxonase; sulfhydryl groups; total, reduced, and oxidized glutathione.

NOx levels were indirectly assessed via plasma nitrite levels based on an adaptation of the method described by Navarro-Gonzalez et al.^6^

Plasma AOPP was quantified based on the method described by Witko-Sarsatet al.^7^ The test was used to measure protein oxidation. AOPP reaction reads were captured on a Thermo Spectronic^®^ Helios-α spectrophotometer (Waltham, MA, USA) at a wavelength of 340 nm. AOPP levels were expressed in µmol/L of chloramine-T equivalents.

Total PON-1 activity was derived from the rate of phenyl acetate hydrolysis (phenol) based on the method described by Richter, Jarvink Furlong.^8^ The rate of phenyl acetate hydrolysis was determined with the aid of a Perkin Elmer^®^ EnSpire microplate reader (Waltham, MA, USA) at a wavelength of 270 nm measured for four minutes (16 readings with 15-second intervals between them) at a temperature of 25ºC. Activity was expressed in U/mL based on the molar extinction coefficient of phenyl acetate (1.31mMol/Lcm-1).

Protein thiol groups in plasma were assessed with a spectrophotometer as described by Miao-Lin Hu.^9^ Analysis is based on the reaction between 5,5’-dithiobis-(2-nitrobenzoic acid) (DTNB) and a protein sulfhydryl group. Reaction readings were taken with a Thermo Spectronic^®^ Helios-α spectrophotometer (Waltham, MA, USA) at a wavelength of 412 nm. Results were expressed in µM/mg of protein.

Erythrocyte glutathione was quantified based on the method described by Tietze et al. modified by Anderson.^10^^,^ 11 Intra and inter assay coefficients of variation were less than 10%.

### Statistical analysis

The data sets were processed in software program Statistical Package for Social Sciences (SPSS, UK) version 20.0. A confidence interval of 95% and a level of significance of 5% (*p* < 0.05) were set in statistical tests.

Quantitative variables were expressed as mean values ± standard error or median values and interquartile intervals based on whether the data followed a normal distribution. The Kolmogorov-Smirnov test was used to check if the variables followed a normal distribution.

The chi-square test was used to compare between the proportions of patient subsets in terms of race/ethnicity, sex, smoking, alcohol intake, hypertension, diabetes, parathyroidectomy, prescription of non-calcium-based phosphate binders, angiotensin-converting-enzyme (ACE) inhibitors, and/or angiotensin II receptor blockers (ARBs), statins, calcium carbonate or acetate, calcitriol, intravenous iron, and EPO.

Variables with a normal distribution - age, sulfhydryl group, hematocrit, hemoglobin, and creatinine - were compared through Student’s t-test. The Mann-Whitney test was used in the comparison of the following variables: time on hemodialysis, BMI, AOPP, NOx, total, reduced, and oxidized glutathione, Kt/V, PTH, serum iron, ferritin, transferrin, total iron-binding capacity, percent transferrin saturation, serum calcium, serum phosphorus, alkaline phosphatase, albumin, IL-6, waist circumference (WC), mid-upper arm circumference, dose of EPO and CRP.

Oxidative stress variables, AOPP, NOx, sulfhydryl group, and oxidized glutathione presented a trend toward having a correlation with ferritin levels or percent transferrin saturation. Multiple linear regression models were built to analyze the possible impact of these parameters on oxidative stress variables controlled for age, sex, BMI, time on dialysis, hypertension, diabetes, smoking, and use of statins. Only variables with a *p* ≤ 0.20 in bivariate analysis were included in the final model.

## Results

The prevalence of anemia with functional iron deficiency in our study was 36.9%. The patients were divided into two groups after the analysis of test results, one with 54 anemic individuals with FID and a second group with 111 patients with iron overload.

Table 1 shows the demographic, epidemiological, and clinical data of patients divided between individuals with FID and anemic patients with iron overload.

**Table 1:** Demographic, epidemiological, clinical, and workup characteristics of patients with CKD, functional iron deficiency, and iron overload

Variable	Functional iron deficiency (n = 65)	Iron overload (n = 111)	*p*
Females	19 (29.3%)	32 (28.8%)	0.9548
Males	46 (70.7%)	79 (71.2%)	0.9548
Race/ethnlclty			0.3253
White	46 (63.0%)	70 (63.6%)	
Black	10 (13.6%)	30 (272%)	
Brown	6 (8.2%)	8 (72%)	
Yellow	3 (4.1%)	3 (2.7%)	
Age (years) (mean) (95%CI)	55.0 (51.0 - 59.1)	54.5 (21.6 - 574)	0.8230
Time on HD (months) (median) (IQR)	48.0 (12.0 - 75.0)	48.0 (24.0 - 81.0)	0.3992
KtV (median) (IQR)	1.370 (1.210-1.480)	1.390 (1.260-1.530)	0.4788
Creatinine (mean) (mg/dL) (95%CI)	8.83 (8.04-9.62)	9.30 (8.71-9.90)	0.3404
Albumin (median) (g/dL) (IQR)	4.05 (3.80-4.30)	4.20 (4.10-4.50)	0.0020^§^
BMI (median) (IQR)	23.1 (21.0 - 26.1)	25.1 (22.0 - 29.2)	0.0075^§^
Waist circumference (median) (cm) (IQR)	89.25 (82.00-99.75)	99.00 (8725-108.00)	0.0036^§^
Mid-upper arm circumference (median) (cm) (IQR)	26.00 (24.00-29.00)	28.00 (26.00-30.50)	0.0076§
Diabetes	65 (89.0%)	97 (88.2%)	0.8583
Hypertension	58 (89.2%)	97 (874%)	0.9020
Smoking	8 (12.3%)	12 (10.8%)	0.9554
Alcohol drinking	10 (9.2%)	18 (16.2%)	0.8843
Previous PTX	13 (20.0%)	15 (23.1%)	0.3565
Use of calcium carbonate or acetate	37 (56.9%)	6 (62.2%)	0.5990
Use of sevelamer	27 (41.5%)	61 (55.0%)	0.1183
Use of calcitriol	23 (35.4%)	38 (34.2%)	0.8770
Use of ACEi/ARB	38 (58.5%)	55 (49.5%)	0.3238
Use of statins	21 (32.3%)	25 (22.5%)	0.2120
Serum calcium (mg/dL) (median) (IQR)	8.12 (783-8.41)	8.42 (8.21-8.64)	0.0963§
Serum phosphorus (median) (mg/dL) (IQR)	5.70 (4.30-6.60)	5.60 (4.70-720)	0.6097
Alkaline phosphatase (median) (U/L) (IQR)	126.0 (85.0-208.0)	161.0 (101.0-215.5)	0.1604
PTH (median) (pg/mL) (IQR)	326.8 (145.30-7070)	462.7 (165.00-941.35)	0.2561

No statistically significant difference was seen in terms of age, sex, time on hemodialysis, or Kt/V in the studied groups. No statistically significant difference was seen in the use of statins, ACE inhibitors/ARBs, calcium carbonate, calcitriol, or sevelamer between the groups. No statistically significant difference was seen in the proportions of smokers or individuals drinking alcohol, patients with hypertension or diabetes, or individuals previously submitted to parathyroidectomy.

The groups were not statistically different in relation to the outcomes of lab tests for calcium, phosphorus, alkaline phosphatase, PTH, ferritin, or creatinine. Hemoglobin levels were significantly lower in anemic individuals with FID.

Inflammation and oxidative stress parameters were not significantly different when patients with FID were compared to individuals with iron overload (Table 2).

**Table 2:** Levels of hemoglobin and markers of iron metabolism, inflammation, and oxidative stress of patients on hemodialysis with functional iron deficiency and iron overload

Variable	Functional iron deficiency (n = 65)	Iron overload (n = 111)	*p*
Hematocrit (%) (mean) (95%CI)	32.84 (31.47-34.22)	34.66 (33.70-35.62)	0.0288
Hemoglobin g/dL (mean) (95%CI)	11.09 (10.64-11.54)	11.83 (11.52-12.14)	0.0063
Serum iron (median) (µg/dL) (IQR)	4740 (41.00-55.40)	90.70 (68.45-122.70)	< 0.0001§
Ferritin (ng/mL) (median) (IQR)	1010.0 (568.3-1360.0)	1030.0 (739.4-1455.0)	0.2295
% transferrin saturation (median) (IQR)	0.170 (0.150-0.185)	0.310 (0.240-0.481)	< 0.0001§
Transferrin (median) (mg/dL) (IQR)	208.0 (182.4-248.0)	233.3 (209.8-260.2)	0.0100§
TIBC (mg/dL) (median) (IQR)	260.0 (228.0-310.0)	291.6 (262.2-325.2)	0.0100§
Use of parenteral iron	44 (677%)	70 (63.1%)	0.6477
Monthly dose of parenteral iron (mg) (median) (IQR)	400 (200.0-400.0)	200 (200.0-400.0)	0.0027
Monthly dose of (IU)	32.000 (32.000-48.000)	32.000 (24.000-48.000)	0.7846
IL-6 (median) (pg/mL) (IQR)	6.78 (4.54-11.81)	6.96 (3.53-12.02)	0.6270
C-reactive protein (median) (mg/dL) (IQR)	9.10 (3.40-16.10)	6.30 (3.05-12.60)	0.1169
AOPP (µM of chloramine T equivalents) (median) (IQR)	175.28 (142.78-224.52)	18770 (145.33-272.04)	0.2050
NOx (µM) (median) (IQR)	10.43 (8.36-15.16)	11.02(8.08-15.91)	0.7256
Sulfhydryl group (mM/mg of protein) (mean) (95%CI)	265.39 (249.83-280.95)	272.93 (261.47-284.39)	0.4340
Total glutathione (mM/g of Hb)	712 (5.99-8.22)	6.98 (6.17-795)	0.5188
Reduced glutathione (mM/g of Hb)	5.07 (4.37-5.90)	4.96 (4.28-5.64)	0.2786
Oxidized glutathione (mM/g of Hb) (median) (IQR)	0.94 (0.67-1.22)	0.99 (0.83-1.23)	0.7360
Paraoxonase (U/mL) (median) (IQR)	139.77 (105.48-164.76)	146.59 (124.25-175.49)	0.0919

Median values (interquartile interval),

§ Mann-Whitney test

Acronyms: FID: functional iron deficiency, CI: confidence interval, IQR: interquartile range, TIBC: total iron-binding capacity, EPO: erythropoietin, IL-6: interleukin-6, AOPP: advanced oxidation protein products, NOx: nitric oxide metabolites.

Parameters serum iron, transferrin, total iron-binding capacity, percent transferrin saturation, BMI, and serum albumin were statistically different between the groups.

Ferritin and nitric oxide were statistically correlated (Table 3).

**Table 3:** Correlations between ferritin levels and percent ferritin saturation and oxidative stress markers

	Ferritin levels		Percent ferritin saturation		
Variables	Spearman's rank correlation coefficient	*p*	Variables	Spearman's rank correlation coefficient	*p*
AOPP	rS= 0.07	0.32	AOPP	rS=0.14	0.06
NOx	rS= - 0.18	0.01*	NOx	rS=-0.003	0.97
Sulfhydryl group	rS= - 0.13	0.08	Sulfhydryl group	rS=0.04	0.59
Total glutathione	rS= 0.047	0.53	Total glutathione	rS= 0.08	0.26
Reduced glutathione	rS= 0.058	0.45	Reduced glutathione	rS=0.01	0.88
Oxidized glutathione	rS= -0.01	0.88	Oxidized glutathione	rS= 0.13	0.08
Paraoxonase	rS= -0.01	0.88	Paraoxonase	rS=0.09	0.23

Table 4 shows that the studied variables were unable to explain the mild negative correlation observed between ferritin and NOx.

**Table 4:** Multiple Linear Regression: regression coefficients after bivariate analysis (non-adjusted coefficients) and multivariate analysis (adjusted coefficients) of the studied variables and NOx levels in the study population

Variables	Non-adjusted coefficients	*p*	Adjusted coefficients	*p*
	Beta		Beta	
Age	- 0.016	0.84	----	
BMI	0.03	0.67	----	
Sex	0.11	0.13	0.14	0.07
Hypertension	- 0.07	0.39	----	
Diabetes mellitus	- 0.003	0.96	----	
Smoking	0.11	0.16	0.13	0.08
Time on dialysis	- 0.03	0.67	----	
Use of statins	0.085	0.26	----	
Ferritin	0.02	0.78	----	
Percent ferritin saturation	0.002	0.97	----	

## Discussion

This study aimed to verify the presence of increased oxidative stress and inflammation in patients with CKD and FID. FID has been associated with increased inflammation, oxidative stress, and malnutrition.^12^

Data analysis revealed that the levels of markers of inflammation and oxidative stress were similar between the two groups (with or without FID).

However, monthly parenteral iron levels were significantly higher and percent transferrin saturation, serum albumin, hemoglobin, hematocrit, BMI, waist circumference, and mid-upper arm circumference were significantly lower in the group with FID. Although difficult to interpret in anemic patients and individuals with anemia and CKD, the lower serum transferrin levels seen in this study may indicate diminished synthesis of the protein in question, a marker of nutritional status in children, patients in postop care, patients on parenteral nutrition, and patients with CKD, to name a few.^12^^,^ 13

The lower serum albumin levels seen in the group of patients with FID, along with the lower levels of hemoglobin and hematocrit and smaller mid-upper arm circumference seem to indicate that, in the absence of a difference in parameters of inflammation and oxidative stress between the two groups, the definition of FID used in this study revealed an association between FID and type 1 malnutrition.

Classical studies on the synthesis of hemoglobin and hemeprotein, whose primary role is to transport oxygen from the lungs to tissues, revealed the importance of having proper serum amino acid levels for the synthesis of the polypeptide chains of the globin chains present in this protein.^14^

The fact that patients with FID were prescribed higher monthly doses of iron than individuals without FID is a concern and a reflection of a possible error in the prescription of iron therapy, since patients in this group have high serum ferritin levels and should therefore not receive higher parenteral doses of iron than anemic patients with some degree of absolute iron deficiency on account of the risk of hemosiderosis. The regimen prescribed to these patients was based on the guidelines for the treatment of anemia in individuals with CKD present in Ordinance 226 issued by the Brazilian Ministry of Health on May 10, 2010.

Although described by a number of authors, the results presented in our study did not support the existence of an association between FID and malnutrition-inflammation complex syndrome.^15^^,^ 16 The broad panel with oxidative stress and inflammation biomarkers, the numerous nutritional parameters, and the definition of FID used in our study^15^^,^ 16 suggested an association with markers of malnutrition, but not increased inflammation or oxidative stress. Differences among study populations including factors such as ethnicity, mutations and polymorphisms in proteins involved in iron metabolism, income, socioeconomic status, nutritional patterns, vitamin supplementation, prevalence of anorexia and depression in dialysis services, anemia management guidelines, and definitions of FID may have contributed to these results.

The cross-sectional design of our study, the relatively small patient sample, and the failure to measure cofactors in hemoglobin synthesis such as vitamin B12 and folic acid were some of the limitations encountered in this study. For purposes of comparison, a previous study enrolling patients from the same dialysis centers involved in the present study revealed that supplementation with routinely recommended vitamins yielded mean acid folic and median vitamin B12 levels within the reference range in 186 patients on hemodialysis.^15^^,^ 16

Intravenous iron therapy requires careful attention, so that doses are decreased and iron overload is prevented in patients on hemodialysis.^17^

Studies with a larger number of patients, more markers of oxidative stress and inflammation, and closer consideration to malnutrition are required to improve the knowledge and management of FID in patients on hemodialysis.

## Conclusion

Our study did not find significant correlations between FID and inflammation and oxidative stress, as suggested in current literature. Our patients had high levels of inflammation and oxidative stress markers probably on account of uremia, and the prevalence of more pronounced inflammation or oxidative stress was 36.9%.

The association between FID and malnutrition verified by the observation of lower BMI, albumin, transferrin, waist circumference, and mid-upper arm circumference in this group seemed to indicate that protein-energy malnutrition might have been a factor.

FID may be caused by the accelerated production of red blood cells induced by the administration of ESA, and by low levels of transferrin secondary to malnutrition and/or lower mobilization of iron stocks in the reticuloendothelial system in contexts of inflammation or infection.^17^ This study aimed to associated FID with states of increased inflammation and oxidative stress, but found a consistent association between FID and protein-energy malnutrition.

A relatively small patient sample, failure to measure folic acid and vitamin B12 levels and red blood cell markers rank among the limitations present in our study. Nevertheless, the study was carried out with patients seen at centers with proper control of water quality, hemodialysis, and hemoglobin levels, aided by a relatively broad panel of biomarkers of oxidative stress and inflammation.

The fact that patients with FID were prescribed significantly higher monthly doses of iron than individuals without FID is a concern. This finding, along with the consideration that the guidelines issued by the Brazilian Ministry of Health ban the use of EPO when percent transferrin saturation is less than 25% even when ferritin levels are above 500 ng/dL, raises questions over the recommendations in effect and may open room for a review of the Brazilian guidelines, since overprescription of iron therapy may lead to hemosiderosis.

According to Ordinance 226 issued by the Ministry of Health, treatment with parenteral iron must be discontinued temporarily when percent transferrin saturation is greater than 50% or serum ferritin is above 800 ng/dL or higher than 1200 ng/dL in patients requiring epoetin alfa doses greater than 225IU/kg/week or 22,500IU/week. After serum ferritin levels have returned to 500 ng/dL or 800 ng/dL in patients requiring high doses of EPO or percent transferrin has decreased to less than 50%, parenteral iron can be restarted at half the previous dose.

Brazilian guidelines validate the indiscriminate use of parenteral iron therapy, since they allow it in patients with low percent transferrin saturation regardless of the presence of high ferritin levels, thus explaining the high doses of parenteral iron prescribed to the patients with FID.

The most recent international guideline (KDIGO 2012) states that parenteral iron must be discontinued with ferritin levels > 500 ng/dL, while the Brazilian guideline contained in Ordinance 226 issued by the Ministry of Health permits the use of parenteral iron in patients with ferritin levels of 500-1200 ng/dL.

Our study indicated the need to review the Brazilian guidelines and calls for stricter protocols for prescriptions of parenteral iron.
